# α5β1 Integrin-Fibronectin Interactions Specify Liquid to Solid Phase Transition of 3D Cellular Aggregates

**DOI:** 10.1371/journal.pone.0011830

**Published:** 2010-07-29

**Authors:** Carlos E. Caicedo-Carvajal, Troy Shinbrot, Ramsey A. Foty

**Affiliations:** 1 Department of Biomedical Engineering, Rutgers University, Piscataway, New Jersey, United States of America; 2 Department of Surgery, University of Medicine and Dentistry, New Jersey-Robert Wood Johnson Medical School, New Brunswick, New Jersey, United States of America; The University of Akron, United States of America

## Abstract

**Background:**

Tissue organization during embryonic development and wound healing depends on the ability of cells on the one hand to exchange adhesive bonds during active rearrangement and on the other to become fixed in place as tissue homeostasis is reached. Cells achieve these contradictory tasks by regulating either cell-cell adhesive bonds, mediated by cadherins, or cell-extracellular matrix (ECM) connections, regulated by integrins. Integrin α5β1 and soluble fibronectin (sFN) are key players in cell-ECM force generation and in ECM polymerization. Here, we explore the interplay between integrin α5β1 and sFN and its influence on tissue mechanical properties and cell sorting behavior.

**Methodology/Principal Findings:**

We generated a series of cell lines varying in α5β1 receptor density. We then systematically explored the effects of different sFN concentrations on aggregate biomechanical properties using tissue surface tensiometry. We found previously unreported complex behaviors including the observation that interactions between fibronectin and integrin α5β1 generates biphasic tissue cohesion profiles. Specifically, we show that at constant sFn concentration, aggregate cohesion increases linearly as α5β1 receptor density is increased from low to moderate levels, producing a transition from viscoelastic-liquid to pseudo viscoelastic-solid behavior. However, further increase in receptor density causes an abrupt drop in tissue cohesion and a transition back to viscoelastic-liquid properties. We propose that this may be due to depletion of sFn below a critical value in the aggregate microenvironment at high α5β1 levels. We also show that differential expression of α5β1 integrin can promote phase-separation between cells.

**Conclusions/Significance:**

The interplay between α5-integrin and sFn contributes significantly to tissue cohesion and, depending on their level of expression, can mediate a shift from liquid to elastic behavior. This interplay represents a tunable level of control between integrins and the ECM that can influence tissue cohesion and other mechanical properties, which may translate to the specification of tissue structure and function. These studies provide insights into important biological processes such as embryonic development, wound healing, and for tissue engineering applications.

## Introduction

The process of tissue self-assembly and its molecular and physical determinants has been a topic of intensive investigation over several decades. The ability of mixtures of embryonic cells to sort-out from one another has been compared to the breaking of a dispersion or emulsion of two immiscible fluids. This liquid-like behavior underlies the theoretical framework, codified by the Differential Adhesion Hypothesis (DAH), to explain how, when dissociated and co-aggregated, cells of two different embryonic tissues re-assemble to adopt their normal histological patterns. The DAH attributes the “sorting-out” behavior of mixtures of embryonic cells as they self-assemble to differences in their strengths of intercellular adhesions, expressible as tissue surface tension [1].

Studies on the molecular determinants of surface tension have revealed a role for both direct cell-cell cohesion, as mediated by cadherins [2], [3], and indirect cell-ECM adhesion, as mediated by the interaction of integrins and fibronectin [4]. Understanding with certainty how these adhesion systems combine to give rise not only to surface tension, but also to other tissue mechanical properties, has been impeded by the multiplicity of factors involved in regulating adhesion. Cells can interact through direct cell-cell effects alone, a mixture of cell-cell and cell-ECM effects, or strictly by cell-ECM effects. Furthermore, these modes of interaction depend on factors such as the level of expression of surface receptors and their cytoplasmic regulators, and the amount and type of extracellular matrix in the microenvironment. Whereas several recent studies have addressed the role of cadherins as determinants of tissue surface tension, few have directly explored the influence of varying both α5β1 receptor density and soluble fibronectin (sFn) concentration on tissue mechanical properties.

Measurements of the surface tension of aggregates of mouse fibroblast L-cells genetically engineered to express cadherin at various levels, and in which those cadherins are effectively the only source of cohesion, describe a linear relationship between surface tension and cadherin expression [2]. However, tissue cohesion is not exclusively mediated by cadherins. Other adhesion systems also contribute to mechanical properties. In a 3D tissue-like context, the ECM may act as a cellular cross-linker, indirectly gluing cells together through integrin-ECM bonds. This concept is supported by studies in which integrin α5-null Chinese hamster ovary cells transfected to express high levels of α5 integrin formed spherical aggregates only in the presence of exogenous fibronectin (Fn) [4], [5], [6]. Indeed, on a molecule per molecule basis, such aggregates were more cohesive than CHO aggregates expressing comparable levels of cadherin. In order to fully support aggregate formation and cohesion, adequate Fn matrix (FnMA) assembly is required. Accordingly, the relationship between the expression of α5β1, sFN concentration, and tissue surface tension may not be as simple as that described for cadherin-mediated cohesion and is a question of investigation in the current study.

Various methods have been developed to measure the surface tension of an aggregate. A common method used, tissue surface tensiometry (TST), applies an external force to an aggregate cultured under physiological conditions, and measures the resistance by the aggregate to the compressive force and changes in aggregate geometry. TST studies over the past 10 years have revealed that aggregates, upon compression, exhibit both visco-elastic solid and liquid-like behaviors, depending on the time-frame over which the measurements are made. In the short-term, compressed aggregates behave as elastic solids: cells in the interior of such aggregates deform upon application of a compressive force and remain deformed until the force is removed, whereupon, analogous to a spring, they rapidly assume their original shape. In contrast, cells in aggregates with viscoelastic-liquid properties, under comparable compressive force, take much longer to regain their original shape as cells slide past one another and overcome the arising friction [7], [8], [9]. These earlier studies revealed that embryonic tissues can not only change their mechanical properties, but can also exhibit a shift in behavior from liquid-like to elastic solid as a consequence of the amount of time in culture and their state of differentiation. For instance, fragments of embryonic chick myocardium cultured for up to 5 days exhibit purely liquid-like behavior. After 5 days in culture, however, the apparent surface tension markedly increases and aggregates then behave almost completely as elastic solids [9]. This change in apparent surface tension and shift in behavior has also been demonstrated during amphibian gastrulation and neurulation. For example, the surface tension of frog deep ectoderm just underlain by the archenteron roof is significantly greater than that of not-yet-underlain deep ectoderm [10]. This suggests that a differentiation-associated shift in surface tension may be responsible for the envelopment of the neural plate.

The transition from liquid to elastic solid has been attributed to ECM deposition that essentially locks cells in place. During the process of convergent-extension, fibronectin deposited on both the inner and outer surfaces of the mesoderm progressively thickens and remodels during the later stages of neurulation. This is thought to contribute significantly to tissue stifness, increasing its capacity to resist bending or kinking under compression, and therefore its ability to push during extension [11], [12], [13]. However, the remodeling process may involve not only changes in the amount of sFn in the microenvironment, but perhaps also an associated change in α5β1 integrin expression and function. This has yet to be empirically demonstrated or quantified. Here, we explore the interplay between α5 integrin expression, sFN concentration, and Fn matrix assembly in cellular aggregates whose cohesion is predominantly mediated by integrin-Fn interaction. Using a simple 3D *in vitro* model, we examine how this interplay influences aggregate mechanical properties, and whether, like cadherin-cadherin interaction, integrin-Fn interaction can also give rise to phase separation between cells. Through this work, we seek to improve the quantitative understanding of complex behaviors of cellular aggregation and organization.

## Results

### Characterization of α5β1 integrin expression by CHO clones

**Figure 1:**
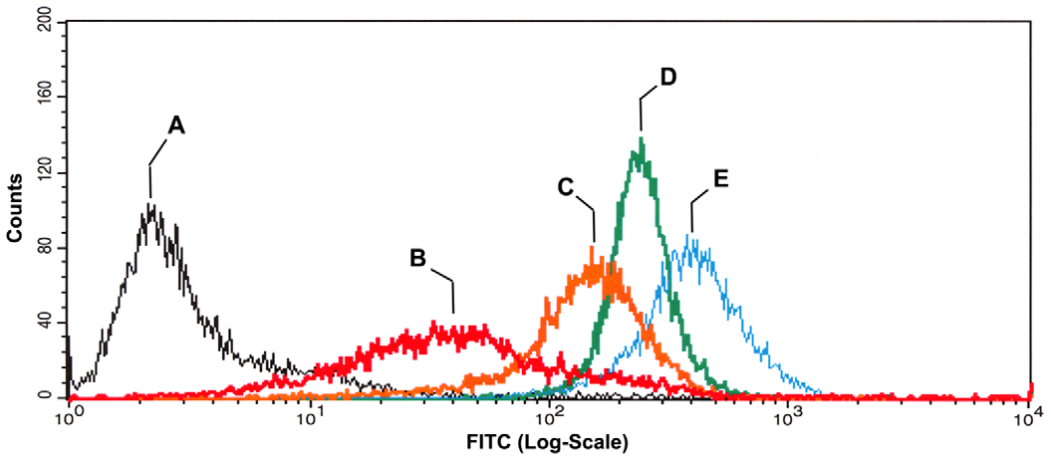
Flow cytometry analysis of α5β1 expression by selected clones. Cells were incubated in hanging drop culture to generate 3D aggregates. Single cell suspensions were then assayed for α5β1 integrin expression by flow cytometry. The relative levels of receptor expression for α5-transfected clones (B–E) were compared with that of the parent cell line transfected with an empty vector control (A). Four distinct populations expressing low (B), mid (C), high (D) and very high (E) levels of α5β1 were expanded and used for all subsequent experiments.

The Chinese hamster ovary cell line CHO-B2 does not express α5 integrin but expresses normal levels of the β1 subunit [14]. The CHO-pCDNA3 line was used as a negative control. Figure 1 shows the fluorescent characteristics of 4 distinct cell populations, derived from cells incubated in hanging drop culture, expressing different levels of α5β1 integrin, from Low (B), to Mid (C), and to High (D), and to highest (E), relative to the empty vector control (A). There is an overall increase in α5β1 expression ranging from 10–100-fold between the empty vector control clone and clones B and E. These clones were then tested for their ability to assemble a fibronectin matrix.

### Characterization of fibronectin matrix assembly as a function of α5β1 expression and soluble Fn concentration

**Figure 2:**
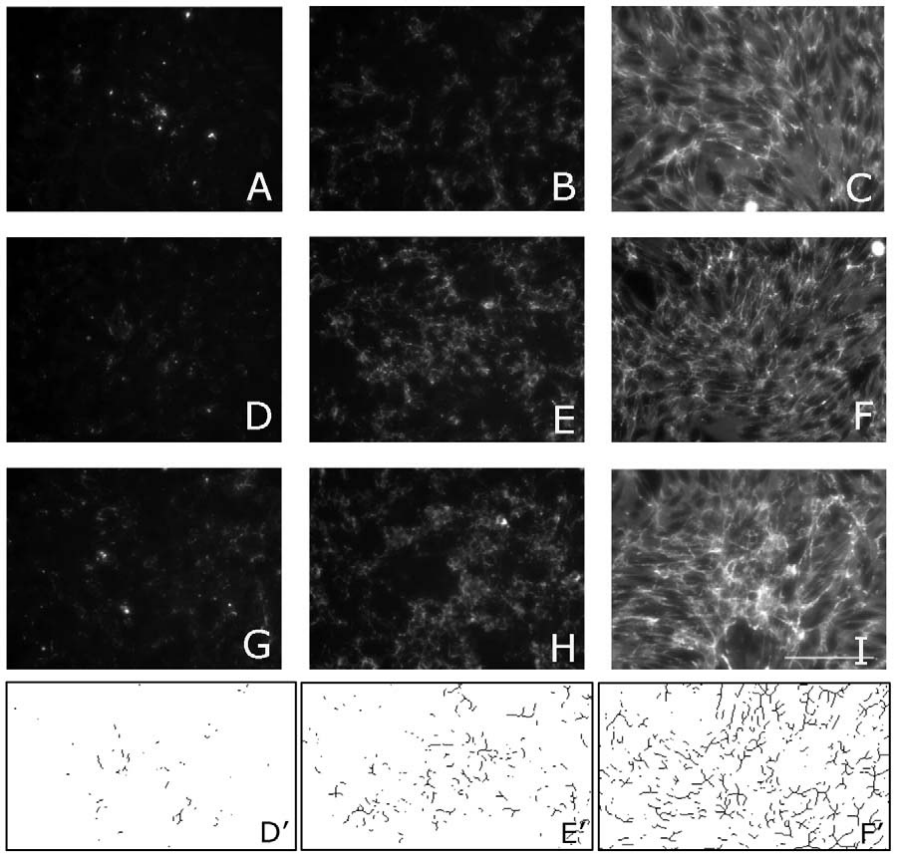
Assessment of Fn matrix assembly by immunofluorescence. Fn matrix assembly by clones expressing low (A–C), High (D–F), and very high (G–I) levels of α5β1 integrin. Clones expressing different levels α5β1 receptor were plated at a density of 5×10^5^ cell/ml. Fibronectin immunostaining was carried out after 2 days in Fn-depleted tissue culture medium (A, D, G) or medium containing 30 µg/ml (B, E, H) or 300 µg/ml (C, F, I) soluble fibronectin. The assembled matrix becomes qualitatively more fibrous as a function of increasing receptor expression and soluble fibronectin concentration. Scale Bar 100 µ.

Interaction between sFn and its receptor drives polymerization of fibronectin dimers into insoluble fibronectin fibers in a process termed fibronectin matrix assembly (FnMA). We assayed each of the clones for FnMA by immunofluoresence. Fig. 2 is a matrix of results showing the interplay between α5β1 receptor expression, sFn concentration, and FnMA. Fig. 2 shows that, in general, increasing sFn concentration gives rise to a denser and more fibrous matrix. We analyzed the images in Fig. 2 by thresholding each image then applying watershed and skeletonization filters using ImageJ analysis software. The rendered images were then subjected to particle analysis. Figs. 2D'–F' are examples of rendered images used to quantify fiber size and fiber density for clone H. Table 1 shows that for each clone, fiber size increases as more sFN is added. Statistical analysis of the data by two-way ANOVA and Bonferroni's post-hoc test describe a strong relationship between fiber length and sFn concentration (p<0.001). Interestingly, there appears to be no statistical difference in fiber size as a function of receptor expression (p = 0.225). Similarly, fiber density correlates strongly with sFn concentration (p<.0001) but not with receptor expression (Table 2).

**Table 1:** Fibronectin fiber size as a function of sFn concentration and α5β1 receptor expression.

		Fiber Size (µm)	
sFN	0 µg/ml	30 µg/ml	300 µg/ml
L	6.5±1.1	15.3±1.6	22.9±2.6
H	5.3±0.8	13.9±1.3	21.1±1.8
HH	5.8±0.7	10.6±1.1	18.2±2.1

**Table 2:** Fibronectin fiber density as a function of sFn concentration and α5β1 receptor expression. mg.v.  =  mean grey value.

		Fiber Density (m.g.v)	
sFn	0 µg/ml	30 µg/ml	300 µg/ml
L	0.96±2.3	7.6±2.9	11.8±4.4
H	0.69±1.9	9.5±2.3	16.2±3.9
HH	0.93±3.6	6.5±2.7	14.1±3.9

### Liquid and elastic solid behavior by α5β1 integrin-expressing clones

**Figure 3:**
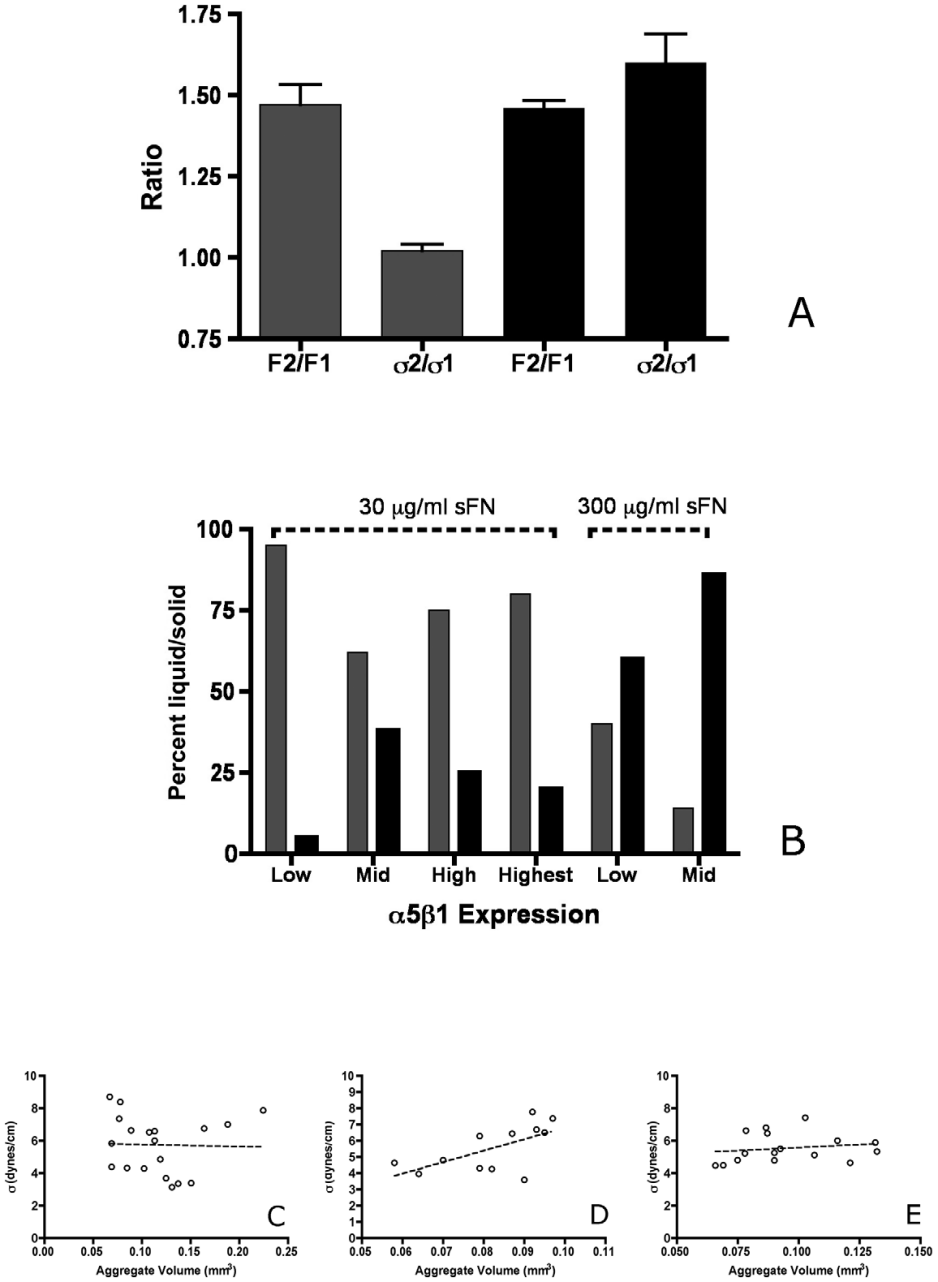
Evaluation of aggregate mechanical properties. α5β1 integrin-Fn interaction can elicit both viscoelastic liquid and elastic solid behavior by compressed aggregates (A). Here we show that for CHO cells transfected to express Mid levels of α5 integrin and incubated in 30 µg/ml sFn, some aggregates exhibit liquid-like behavior, where σ_2_/σ_1_ = 1 and is significantly less than F_2_/F_1_ (grey bars), whereas others exhibit elastic behavior, where the ratio of σ_2_/σ_1_approaches that of F_2_/F_1_ (black bars), (two-proportion z test, α = 0.05). Relative percentage of liquid and elastic aggregates as a function of α5β1 receptor expression and sFn concentration (B). Aggregates incubated in 30 µg/ml sFn, irrespective of α5β1 expression, displayed a preponderance of liquid behavior, (grey bars) whereas those incubated in 300 µg/ml sFn behaved predominantly as elastic solids (black bars). Each data set contained 16–20 aggregates. Linear regression analysis of surface tension vs. volume for aggregates expressing various levels of α5β1 integrin. Figures 3C,D, and E represent clones CHO-X5C5 L, H, and HH, respectively. The circles represent data of individual aggregates within each data set. The line is a regression analysis. The data set is for aggregates displaying liquid-like behavior and was generated for aggregates compressed in standard tissue culture medium. The surface tension of aggregates remained relatively constant over a 5-fold range in volume. Linear regression analysis generated correlation coefficient (r^2^) values of 0.0009 (Fig. 3C), 0.38 (Fig. 3D), and 0.03 (Fig. 3E) for α5β1 (L), α5β1 (H) and α5β1 (HH), respectively. These r^2^ values indicate that no statistically significant correlation exists between σ and volume (C–E).

For all clones, application of a compressive force revealed both liquid and elastic solid behavior by aggregates in each data set. We differentiate between liquid and elastic solid behavior by describing the relationship between initial applied force at a first compression (F_1_) and a greater second compression (F_2_), and the corresponding surface tensions (σ_1_ and σ_2_) measured at shape and force equilibrium for F_1_ and F_2_. We show that in aggregates expressing Mid levels of α5β1 integrin and incubated in 30 µg/ml sFN, comparison between force ratio F_2_/F_1_ and σ_2_/σ_1_ shows that the ratio of σ_2_/σ_1_ approaches 1, whereas the ratio of F_2_/F_1_ (when F_2_ is greater than F_1_) is significantly greater than σ_2_/σ_1_, confirming that these aggregates behave in a typically liquid-like manner. Other aggregates within this same data set exhibited elastic-solid behavior. In this case, the ratio of σ_2_/σ_1_ was similar to the ratio of F_2_/F_1_ (two-proportion z test, α = 0.05, Fig. 3A).

### The ratio of aggregates displaying liquid:elastic behavior changes as a function of α5β1 expression and sFn concentration

Figure 3B shows the relative fractions of viscoelastic-liquid and viscoelastic-solid aggregates in data sets of 16–20 aggregates, as a function of α5β1 receptor expression and soluble fibronectin concentration. The gray bars represent the fraction of aggregates that behave as viscoelastic-liquids, whereas the black bars represent the fraction of aggregates that behave as viscoelastic-solids. We see a clear transition in the liquid-like to elastic-solid states when sFn concentration is increased from 30 to 300 µg/ml. For example, 95% of aggregates of CHO- α5β1(Low) incubated in 30 µg/ml sFn display viscoelastic-liquid behavior, however when aggregates of the same clone were incubated in the presence of 300 µg/ml sFn, only 40% displayed liquid behavior. More striking still, 60% of aggregates expressing Mid levels of α5β1 integrin when incubated in 30 µg/ml sFn exhibit liquid-like behavior, whereas this percentage is reduced to only 15% when the same clone is incubated in 300 µg/ml sFn. The relationship between α5β1 receptor expression level is surprisingly complex even at standard sFn concentration. In aggregates expressing Low to Mid levels of α5β1 integrin, the fraction of aggregates that behave as viscoelastic-liquids decreases from 95% to 62%, however in aggregates ranging in expression levels from Mid (M) to high (H) and highest (HH), this fraction increases from 62% to 75% and 80% respectively.

### For aggregates displaying liquid-like behavior, surface tension is independent of volume

The surface tension of a liquid aggregate is known to be independent of its size [8], [9]. This property is a simple representation of the applicability of the Young-LaPlace surface tension equation to simple fluids. In contrast, elastic solids demonstrate a direct relationship between size and surface tension as per linear elasticity theory. We explored the relationship between volume and surface tension for aggregates in which the ratio of σ_2_/σ_1_ = 1 for various levels of α5β1 integrin expression. At low (3C), high (3D), or highest (3E) levels of integrin expression, surface tension appears to be independent of aggregate volume since linear regression analysis generated correlation coefficient values well below those required to establish a significant relationship between these two parameters. Accordingly, such aggregates display liquid-like behavior and made it possible to explore the relationship between α5 receptor expression and surface tension.

### Surface tension measurements of aggregates displaying liquid-like behavior as a function of α5β1 receptor expression

**Figure 4:**
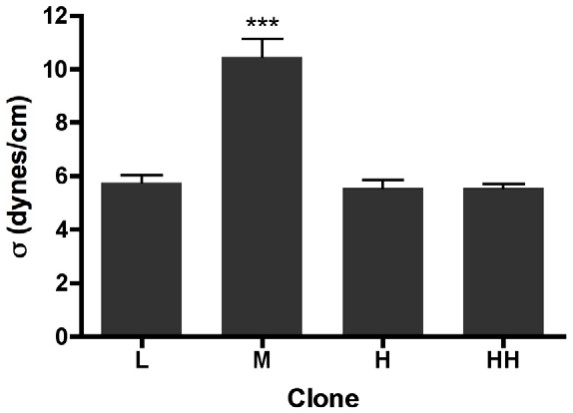
Surface tension of aggregates of α5β1 integrin-expressing clones. Surface tension values of aggregates displaying liquid-like behavior were plotted as a function of α5β1 receptor expression. Aggregates expressing Mid levels of α5β1 integrin possess a higher surface tension than all other clones (ANOVA, p<0.0001, Tukey's MCT, p<0.001). Surface tension appears to increase as a function of receptor expression but only for aggregates expressing low (L) to mid (M) levels of α5β1 integrin. Higher level expression of the receptor results in aggregates whose surface tensions are not significantly different than for that measured for low-expressing aggregates. Data sets contained 20–38 aggregates, representing 40–76 compressions.

Previous studies have shown that for cadherin-based adhesion, the relationship between surface tension and receptor expression is essentially linear. We set out to determine whether a similar relationship exists for integrin-Fn based adhesion. To do so, we calculated σ for aggregates exhibiting liquid-like behavior for each level of receptor expression. As can be seen in Fig. 4, tissue surface tension of the Mid-level expressing clone is significantly higher than that of the other three clones (ANOVA, p<0.0001, Tukey's Multiple Comparisons test, p<0.001). Surface tension increases from 5.7±0.3 to 10.4±0.7 dynes/cm as receptor expression levels increase from Low to Mid. However, a further increase in α5β1 expression to high (H) and highest (HH) results in a paradoxical drop in surface tension back to 5.5±0.3 dynes/cm and 5.6±0.16 dynes/cm, respectively. These data demonstrate that the relationship between α5β1 integrin-based adhesion and aggregate surface tension is more complex than that for cadherin-based cohesion, and suggest that coaction between receptor density and sFn concentration provides dynamic control of tissue mechanical properties. We next set out to explore the physical and molecular mechanisms underlying the biphasic nature of the α5 integrin/surface tension relationship.

### Kinetic compaction profiles as a function of cell number

**Figure 5:**
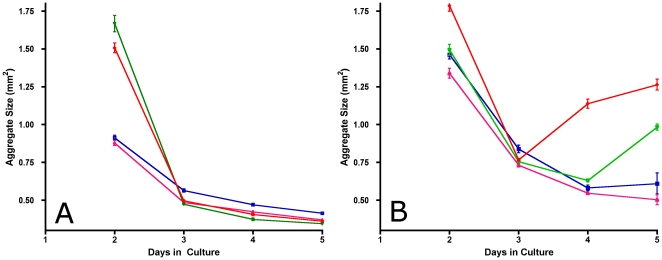
Aggregate size as a function of cell number and time in culture. Compaction assays were performed in which cells for all clones were placed in hanging drop culture at cell seeding densities of 25,000 (A) and 50,000 (B) cells per aggregate and a sFn concentration of 30 µg/ml. Size measurements were initiated on day 2 and repeated daily up to day 5. Each data point represents average size calculated from 10 aggregates per time point for clones expressing Low (blue line), Mid (pink line), High (green line) and Highest (red line) levels of α5β1 integrin.

One possible explanation as to the biphasic nature of the α5β1/surface tension relationship is that in the presence of limiting sFn, α5β1 receptors bind and deplete the available matrix to a point that no longer supports matrix assembly. We tested this possibility by performing aggregate compaction assays using hanging drops containing either 25,000 or 50,000 cells and measuring the rate of compaction of aggregates over a 5-day time period. As can be seen in Fig. 5A, aggregates containing 25,000 cells decrease in size monotonically over time. The compaction profiles displayed have three characteristic features: a) the compaction at day 2 is much slower for aggregates expressing Low and Mid levels of α5β1 integrin than for aggregates expressing high (H) and highest (HH) levels, with approximately a 2-fold difference in compaction rate between the two groups (Two-way ANOVA, p<0.0001, Bonferroni's MCT, p<0.001), b) by day 3, all aggregates have compacted significantly, and c) thereafter, compaction profiles level off for all cell lines, reaching similar aggregate size in all cases. For aggregates containing 50,000 cells (Fig. 5B), the compaction profiles were essentially similar between days 2 and 3, however on day 4, aggregates expressing higher levels of α5β1 integrin decompacted and were significantly larger than was observed for aggregates containing 25,000 cells (p<0.0001). These results are consistent with the hypothesis that higher integrin expression results in increased usage of available sFn, causing the highest expressing cell lines to deplete the available sFn, which leads to aggregates that first decrease in size, then increase as sFn is depleted from the medium.

### sFn depletion and its assembly into a fibrous matrix by aggregates expressing different levels of α5β1 receptor

**Figure 6:**
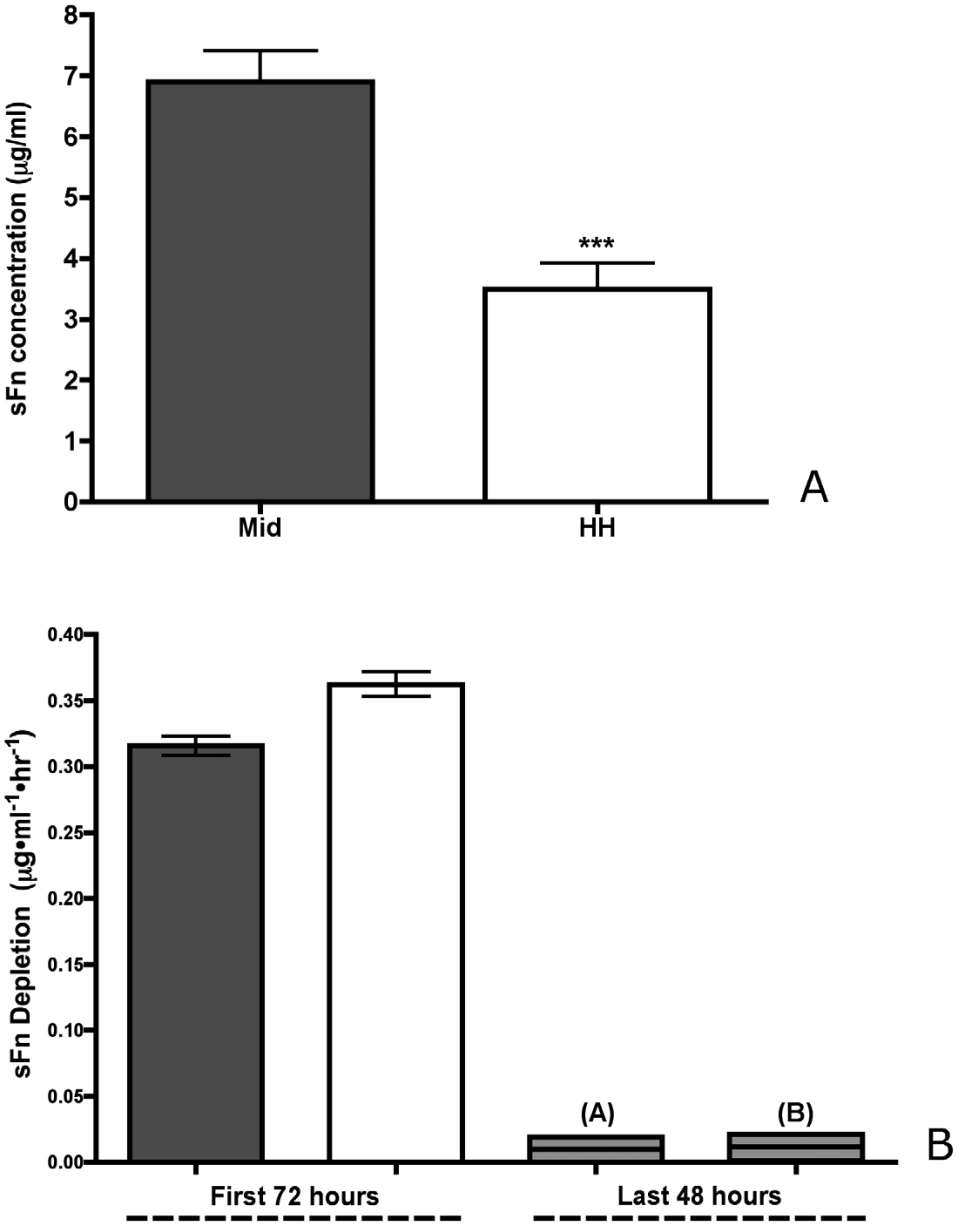
sFn depletion as a function of α5β1 expression. (7A) Twenty-five thousand cells of the Mid (grey bars) and HH (white bars) clones were incubated in 30 µg/ml sFn in hanging drop culture for 3 days, at which time the amount of sFn remaining in each hanging drop was assayed by ELISA. Cells expressing Mid levels of α5β1 integrin depleted sFn at a slower rate than did cells expressing higher levels (Student t-test, p = 0.0005). (7B) For the first 72 hours, the rate of depletion of sFn from the hanging drop is significantly higher for HH aggregates than for Mid (unpaired t-test, p<0.05). After 72 hours, the rate of sFn utilization drops markedly and is the same for both cell lines (unpaired t-test, p>0.05).

We measured the concentration of sFn after 72 hours of incubation in hanging drop cultures originally containing 30-µg/ml sFn. Fig. 6A compares the effect of α5β1 expression (from M to HH) on sFn concentration. sFn concentration in hanging drops of HH aggregates is significantly lower than sFn concentration in aggregates expressing lower levels of the receptor (unpaired t-test, p = 0.0005). We also compared the rate of sFn utilization (µg/ml·hr) of α5β1(M) and α5β1(HH) aggregates, over a 5-day period. Fig. 6B shows that the rate of sFn depletion has two time-scales: for the first 72 hours, the rate of depletion is significantly higher for HH aggregates than for Mid (unpaired t-test, p<0.05). This appears to correlate with the differences in aggregate compaction observed in Fig. 6. For the last 48 hours, the overall rate of sFn utilization drops markedly and appears to be equivalent for both cell lines (unpaired t-test, p>0.05). We next asked whether fibronectin matrix assembly can also be influenced by overexpression of receptor in limiting sFn concentration, as this could explain the mechanism underlying decompaction and decreased aggregate surface tension.

### Fibronectin matrix assembly in 3D aggregates as a function of α5β1 receptor expression

**Figure 7:**
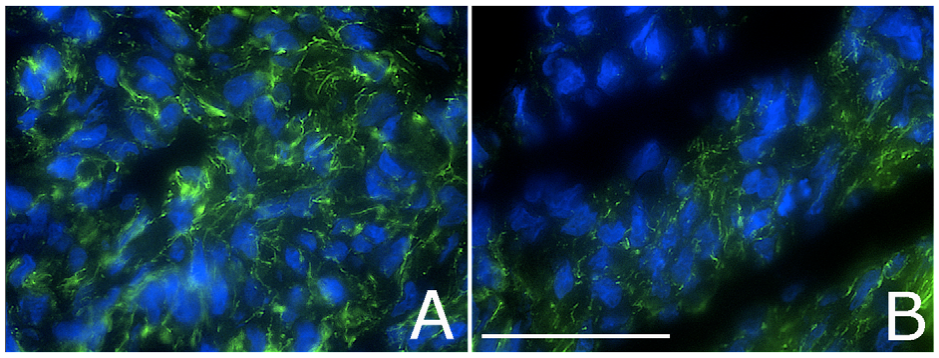
Fibronectin matrix assembly in 3D aggregates as a function of α5β1 receptor expression. Representative immunostained tissue sections from aggregates containing 25,000 cells and expressing either Mid (A) or HH (B) levels of α5β1. Images are of aggregates collected after 3 days of incubation. Fibronectin matrix is labeled green and nuclei are blue. The scale-bar is 50 µ.

**Figure 8:**
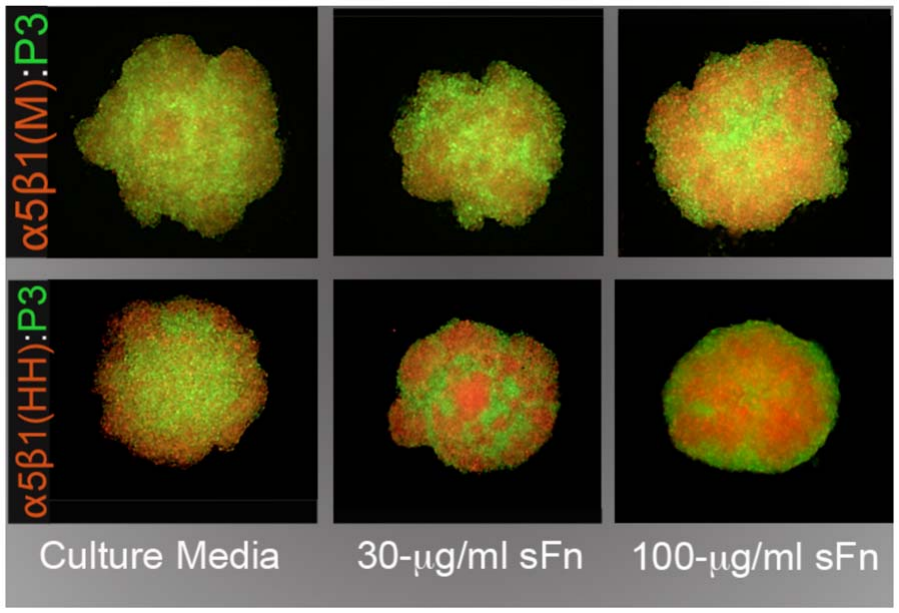
α5β1 integrin-mediated cell sorting. Differentially stained cells of M (red) or HH (red) clones sort out from CHO-P3 cells (green) to assume an internal position. This sorting behavior appears to become more apparent as both α5β1 expression and sFn levels increase.

Figure 7 depicts immunostained frozen sections of aggregates containing 25,000 cells expressing either Mid (Fig. 8A) or HH (Fig. 8B) levels of α5β1 integrin and incubated in 30 µg/ml sFn. Aggregates expressing Mid levels of sFn appear to assemble a more fibrous matrix (A) than aggregates expressing much higher levels of the receptor (B). Interestingly, HH aggregates incubated in 30 µg/ml sFn as 2D culture (Fig. 2H) also generate a less dense matrix. This was confirmed by image analysis of areas in each image where fluorescence signal was detected. On average, fluorescence intensity of aggregates composed of α5β1 M cells was higher than that of α5β1 HH. This suggests that high levels of receptor expression may effectively reduce the overall amount of fibrous matrix, interfere with the establishment of a global, interconnected ECM, and decrease the degree of cross-linking needed to maintain strong aggregate cohesion.

### Sorting behavior between α5β1-expressing clones and control CHO-P3 cells as a function of receptor expression and sFn concentration

We tested whether α5β1 integrin-fibronectin interaction can induce cell sorting between clones expressing different levels of α5β1 integrin and CHO-P3 cells and the effects of fibronectin concentration on the process. Figure 8 shows the different rearrangement behaviors of α5β1 M and HH cells, labeled red, mixed with CHO-P3, labeled green. The equilibrium configurations adopted after 24 hours in HD culture were assessed by epifluorescence microscopy. As can be seen in Fig. 8, phase separation between clones and P3 cells is dependent on receptor expression and amount of sFn. Mixed cells exhibited progressive clustering of red CHO- α5β1(M) cells within a field of green CHO-P3 cells. This clustering increased as the amount of soluble fibronectin in the medium increased. At 100 µg/ml sFn α5β1 M cells generally adopted an internal position relative to P3 cells. This configuration becomes more evident when α5β1 HH cells are mixed with P3. At low soluble fibronectin concentration, the α5β1(HH)(red) population appears to initially adopt an external position (peripheral segregation) with respect to the P3 population(green). As the concentration of fibronectin increases to 30 µg/ml then to 100 µg/ml, α5β1(HH) cells progressively come to occupy a more central position.

## Discussion

Cell-cell cohesion and cell-ECM adhesion underlie a broad range of normal morphogenetic and disease processes including embryonic development, wound healing, and malignant invasion. These two key adhesive mechanisms are mediated in large part by the expression and function of cadherins and integrins. Cadherin-based cell-cell cohesion arises as a consequence of direct cadherin to cadherin interaction. In contrast, integrin-based cell-cell cohesion is indirect and in large part mediated by the presence of extracellular matrix, a component whose existence adds a layer of complexity, and more importantly, a potential mediating effect, on the bulk-cohesive property of a tissue. Accordingly, integrin-mediated and cadherin-mediated adhesion operate through different mechanisms and with different time scales that may influence their roles during embryonic development and other biological processes such as wound healing and malignant invasion. In this study, we explore the interplay between α5β1 integrin expression and soluble fibronectin concentration using a simple in vitro 3D hanging drop model.

In a 3D aggregate, the ECM is not only an important source of cell-substratum adhesion for cell motility and shape change, but may also act as a cellular cross-linker, indirectly “gluing” cells together through integrin-ECM bonds. Previous studies showed that Chinese hamster ovary (CHO) cells, transfected to express high levels of α5 integrin, formed spherical aggregates only in the presence of soluble Fn [4]. Aggregate formation was also shown to be dependent on the ability of cells to assemble FN into an insoluble matrix [5]. Interestingly, integrin-based cohesion was found to be, on a molecule per molecule basis, more effective at mediating strong tissue cohesion than N-cadherin, a more classic adhesion system that is more commonly identified as a significant source of cohesion of cells in tissues. In those studies, measurement of aggregate cohesion was performed under conditions in which α5 integrin expression and the amount of sFn were kept constant. Accordingly, nearly all aggregates in any particular data set displayed liquid-like behavior. Here we varied both α5 integrin expression levels and the amount of available sFn. In so doing, we observed differences in FNMA, aggregate cohesion, and shifts in mechanical properties from liquid to elastic solid, such that in some data sets, nearly all aggregates displayed elastic solid-like behavior. We assessed this shift in material properties by determining whether aggregates, when compressed, obeyed Hooke's Law.

We found that when α5 expression is increased from low to moderate levels, surface tension increases from 5.5 dynes/cm to 10.4 dynes/cm, but further increase in α5 receptor expression paradoxically caused a drop in surface tension back to those measured for low levels of α5 expression. This was associated with a biphasic transition from viscoelastic-liquid to viscoelastic-solid behavior such that aggregates behaved as viscoelastic-solids for moderate α5 expression levels and liquid-like for either higher or lower expression values. These data are in sharp contrast to those observed for cadherin-based cell-cell cohesion, where studies have demonstrated a near-perfect correlation between receptor expression and surface tension and no associated transitions in tissue mechanical properties. Interestingly, the biphasic nature of the relationship in 3D tissues between receptor expression and surface tension appears to mirror the relationship in 2D between integrin receptor expression and cell migration [15]. That is, an optimal level of integrin expression is required in order to maximize cell motility. In the current study, aggregates expressing either low or high levels of α5 integrin exhibited liquid-like behavior, indicative of an actively motile cell population, whereas cells expressing intermediate levels of integrin became essentially locked in place, behaving as elastic solids. For cells to become locked in place, they must lose the ability to detach from one-another or from the matrix. Accordingly, cells must no longer be able to detach from the matrix by release of integrin-matrix bonds [16] or by fibril breakage [17]. It is likely that in aggregates displaying elastic solid behavior, the fibronectin matrix is stiffer than that of aggregates displaying liquid-like behavior. Matrix stiffness has been shown to significantly influence cell movement in 3D in vitro [18] and in vivo [19].

The biphasic nature of the interaction between α5 integrin expression and sFn levels can be explained by considering the role of sFn in providing the means by which cells become cross-linked through integrin-ECM interaction. Previous studies exploring integrin-mediated tissue cohesion have demonstrated a critical role for fibronectin matrix assembly in generating strong tissue cohesion [4], [5]. In this study, we varied the amount of sFn and showed that at 30 µg/ml, irrespective of α5 expression levels, aggregates behaved predominantly as liquids, whereas increasing the fibronectin concentration 10-fold rendered aggregates, even those expressing low levels of α5 integrin, almost completely elastic. Moreover, we showed that when aggregates composed of 25,000 or 50,000 cells are incubated in hanging drop culture, those containing 25,000 cells tend to compact over a 5-day period irrespective of the level of integrin expression. In contrast, those containing 50,000 cells initially compact over 3-days, but then undergo a significant decompaction as sFn becomes depleted from the microenvironment. This effect was more pronounced for cells expressing higher levels of α5 integrin, and corresponded closely to the time-frame of sFn depletion from the medium in the hanging drops.

During embryonic development, both receptor expression and ligand levels change. Studies have shown that embryonic tissues isolated at different stages of development exhibit significantly different mechanical properties, and that tissues isolated from earlier stages of development can undergo similar changes if extirpated and grown in culture [9]. For example, the cohesivity of chick embryonic heart aggregates was shown to be nearly constant for 4 days in culture, whereupon it nearly tripled when aggregates were incubated for another 3 days [9]. This increase in cohesion was predominantly due to a transition from liquid to elastic solid behavior by aggregates. Changes in mechanical properties can result in phase reversals between tissues, as well as in the elimination of boundaries between different tissue types, and are thought to occur primarily due to changes in the level of fibronectin in the microenvironment [9], [20], [21], [22], [23]. Earlier studies showed that when heart myocyte aggregates, one pre-incubated in normal medium, the other in a urea extract of a fibroblast-conditioned medium, were fused in hanging drop culture, the one pre-incubated in fibroblast-conditioned medium became enveloped by the control aggregate [22], and was blocked by inclusion of GRGDSP peptide [24]. This type of segregation behavior typically occurs between tissues of differing cohesivity [9]. In heart myocytes, envelopment of one tissue by the other was plausibly mediated by the presence of fibronectin in the urea extract of the fibroblast-conditioned medium [25]. By cross-linking cells together, fibronectin would effectively increase the cohesion of the treated myocytes, causing them to internalize relative to their untreated counterparts. Other studies reveal that fibronectin deposition can also stabilize the interface between tissues and prevents invasion of one tissue type by another [24]. More recent studies have shown that ECM formation can become restricted to tissue surfaces and interfaces by a mechanism utilizing surface de-repression of integrin-dependent inhibition of matrix assembly and that this enables self-organization and maintenance of tissue boundaries [26].

Our studies reveal that it may not simply be a matter of ECM deposition, but a quantitative interplay between fibronectin and integrins that collectively regulate tissue mechanical properties. The exact mechanism underlying how this interaction can directly influence tissue properties likely involves integrin activation [27] and the assembly of sFn into an insoluble matrix [28]. Indeed, previous studies have shown that fibronectin matrix assembly (FnMA) is necessary for imparting strong tissue cohesion to aggregates whose primary cohesion mechanism is integrin-based, since CHO cells transfected to express a chimeric integrin which does not support FNMA resulted in flat sheets of cells that failed to round up into spheres [5]. Additionally, fibronectin deposition by these aggregates was punctate. Fibers, when present, were short and tended to extend only locally. We showed that FNMA by aggregates expressing mid levels of α5β1 integrin generated a rich matrix, with fibers extending from cell-cell throughout the aggregate. FNMA by aggregates of cells expressing high levels of α5 integrin was, in contrast, reduced and tended to generate pockets of matrix assembly and shorter fibers. This may explain why such aggregates tend to be of lower cohesion and more liquid-like than aggregates in which the matrix is better developed. A matrix composed of shorter fibers may facilitate cell migration by effectively reducing the apparent viscosity of the tissue as cells may be less globally interconnected.

The mechanism by which a liquid-solid transition emerges appears to be related to that previously described in other problems, for example in polymer pinning [29], and in granular jamming transitions [30]. In these systems, as lengths of polymers or granular stress chains grow, connections become entangled and the scale over which rearrangements can occur becomes vanishingly small. We believe that the same mechanism is likely at work in our cellular system: when the density and length of the fibronectin network exceeds a critical value, cells can no longer move with respect to one another. Since cellular rearrangements are intrinsic to the liquid state, this is manifested as a transition from liquid-like to solid-like tissue rheology. Our study demonstrates that α5β1 interaction can promote phase transition from liquid-like to elastic solid-like states. Increased cell-matrix engagement to a 3D matrix has been demonstrated to significantly influence viscoelastic properties of prostate cancer cells embedded within 3D matrices [31]. The observation that α5β1 integrin is often down-regulated in metastatic cancers, and that re-expression has been shown to rescue a transformed phenotype [32], is consistent with our view that matrix-based transitions between liquid and solid states may play an important role in specifying whether normal or cancer cells remain locked in place or are free to move and interact with other cell types.

Differences in tissue cohesion have been demonstrated to play a critical role in not only establishing compartments and boundaries between tissues, but also in specifying the spatial relationships that give rise to tissue architecture and to the overall body plan. The Differential Adhesion Hypothesis (DAH) was formulated to explain how the cell rearrangement and reorganization observed during the process of tissue self-assembly can be driven by tissue mechanical properties, i.e. cohesion, adhesion and surface tension [33], [34]. While the process is inherently a physical one, it has molecular roots traceable largely to the activities of adhesion systems mediating both cell-cell and cell-ECM interactions [23]. The contribution of cadherins to tissue cohesion, cell rearrangement, and boundary formation during embryonic development is well known. From a molecular perspective, differences in the level of expression of a cell adhesion molecule (cadherin) have been experimentally shown to cause cell sorting [2]. Cells interacting directly through cadherin-cadherin bonds re-arrange to reduce the adhesive free energy of the system. It is as yet unclear how differences in integrin expression levels could drive the cell-sorting process. Several physical models have been advanced to address this question. Matrix-driven translocation [35], mesenchymal condensation [36], and percolation networks [37], have been proposed to explain ECM-based sorting, particularly between mesenchymal tissues (reviewed in [38]). We show that both CHO-X5C5 M and HH sort out from CHO-P3 to assume an internal position. This behavior is in general agreement with the DAH, which would predict that CHO-X5C5 M and HH must be more cohesive than CHO-P3. However, since CHO-P3 cells do not form spherical aggregates, it was not possible to confirm this by directly measuring aggregate cohesion. Accordingly, it is possible that sorting between the α5-null and α5-expressing CHO cells could be mediated by one or more of these mechanisms.

As we demonstrate in Table 2, CHO-X5C5 M and HH have different surface tensions, HH higher than M. When mixed with CHO-P3 cells, CHO-X5C5 HH sort out more efficiently than CHO-X5C5 M cells and in a sFn-dependent manner. These data are relevant since, at least in some cancer settings, down-regulation of α5β1 integrin is a hallmark of malignancy [39]. Accordingly, cancer cells in which α5β1 is down-regulated may be excluded from those expressing the receptor and become effectively a different cell population that may become squeezed away from the tumor mass, assuming a more peripheral position. This could, in principle, place such cells in contact with stromal cells into which they can then invade.

This study showed that the interplay between α5-integrin and sFn can contribute significantly to tissue cohesion and, depending on their level of expression, can mediate a shift from liquid to elastic behavior. In principle, this interplay represents a tunable level of control over tissue mechanical properties that is at least as powerful as that engendered by cadherin interaction. Understanding how interaction between integrins and the ECM influence tissue cohesion and other mechanical properties which may translate to the specification of structure and function will provide insights into important biological processes such as embryonic development, wound healing, and malignant invasion, and may provide new approaches for the control of tissue engineering applications.

## Materials and Methods

### Cell lines

CHO-B2 cells were maintained in Dulbecco's Modified Eagle's Medium (Invitrogen, Carlsbad, CA), supplemented with 10% fetal calf serum (Hyclone, Logan, UT), 1 mM sodium pyruvate, 0.1 mM MEM non-essential amino acids, and 100 µg/ml streptomycin sulfate (Invitrogen, Carlsbad, CA) and incubated at 37°C and 95% air/5% CO_2_. Transfected cells were maintained in the same medium but with 250 µg/ml G418 (Invitrogen, Carlsbad, CA).

### Generation of α5 integrin-expressing cell lines

CHO-B2 cells do not express α5 integrin [14]. Cells were transfected with an expression vector expressing human α5 integrin by electroporation. Approximately 10^6^ cells were suspended in 400 µl of transfection medium (RPMI, 0.1 mM DTT, 10 mM Dextrose). Twenty µg of α5 cDNA were electroporated at 200 volts and 960 µF in a 0.4 cm electroporation cuvette using a Biorad Gene Pulser II apparatus. Transfected cells were plated into 100 mm tissue culture plates, grown for 24 hours, whereupon they were selected in 800 µg/ml of G418 until resistant cells reached 40–50% confluence. Empty vector control cells (PcDNA3 only) were generated using a similar process of transfection and selection. Transfected cells were screened for α5 integrin expression by flow cytometry.

### Flow cytometry and F.A.C.S

G418-resistant cells were detached with trypsin-EDTA (Gibco-BRL, NY), washed three times with ice-cold Hanks' balanced salt solution (HBSS), and incubated with an anti-human α5 integrin antibody (CD49e, PharMingen, CA) at 5 µg/ml on ice for 45 minutes. Cells were again washed with cold HBSS and incubated on ice for an additional 45 minutes with an Alexafluor-488-conjugated goat-anti-mouse secondary antibody (Zymed, San Francisco, CA). Cells expressing α5 integrin were sorted by F.A.C.S. (EPICS ALTRA, Beckman Coulter, FL) and expanded. These cells were then subjected to several rounds of F.A.C.S., using a Beckman-Coulter MoFlo XDP Cell Sorter, each time selecting different fluorescent peaks. Alternatively, cells were subjected to limiting dilution cloning. Four cell lines; CHO- α5 (L), CHO- α5 (Mid), CHO- α5 (H) and CHO- α5 (HH) were generated, each expressing different and distinct levels of α5 integrin. Clones were then re-assessed for α5 integrin expression by flow cytometry, but this time from aggregates in hanging drop culture. Aggregates were collected, pooled, and incubated in 500 µl trypsin-EDTA for 10 minutes at 37°C whereupon 500 µl of FCS and 1 µg/ml DNAse I was added. Aggregates were then gently triturated into a single cell suspension, washed several times in PBS and processed for flow cytometry using a Becton Dickinson FACSCalibur flow cytometer. The parent CHO-P3 cell line was used to establish instrument baseline settings. Ten-thousand cells from each clone were then compared for α5 integrin expression.

### Assessment of fibronectin matrix assembly by immunofluorescence microscopy

For lines expressing different levels α5β1 receptor, cells were plated onto glass coverslips at a density of 5×10^5^ cell/ml in either Fn-free medium (fecal calf serum was depleted of fibronectin by incubation with collagen sepharose [40]), in medium containing 30 µg/ml soluble rat plasma fibronectin (sFn), or in medium supplemented with 300 µg/ml sFn (Calbiochem, Los Angeles, CA). After 24 hours in culture, the near-confluent monolayers were fixed in 4% paraformaldehyde/PBS for 15 minutes at room temperature, then blocked in blocking solution (0.1% bovine serum albumin in PBS) for 30 minutes. After blocking, coverslips were incubated in rabbit polyclonal anti-fibronectin primary antibody (AB6584, AbCam, Cambridge, MA) at a 1∶100 dilution in blocking solution for 45 minutes at RT. After two washes with PBS, coverslips were incubated in a 1∶100 dilution of a goat-anti-rabbit secondary antibody conjugated to Alexa-fluor 488 for 30 minutes at RT. After washing twice with PBS, stained cells were imaged using a Spot color camera (Diagnostic Instruments, Sterling Heights, MI) connected to a MacIntosh G4 computer equipped with IPLab image analysis software. For 3D aggregates, 5 µ-thick frozen sections were mounted on glass slides. Slides were thawed at RT for 10 minutes and air-dried. A Pap pen (Zymed, CA) was used to encircle each section and sections were then sequentially incubated in primary anti-fibronectin antibody and an Alexa-Fluor 488 secondary antibody as described above.

### Image analysis

ImageJ was used to analyze each image in Fig. 2 and 8. Each image was inverted in Adobe Photoshop and opened in ImageJ. Threshold settings were applied uniformly to all images. Watershed and skeletonization filters were then applied. The rendered images were subjected to particle analysis. Two parameters were measured; fiber size represents the relative “length” of each skeletonized fiber, and density represents the mean grey value of the fluorescence intensity of each image.

### Measurement of aggregate cohesivity by tissue surface tensiometry

#### a) Aggregate preparation by the hanging drop method

Aggregates were generated as previously described [4]. Briefly, near-confluent monolayers were dissociated from 10-cm tissue culture plates with trypsin/EDTA (TE). Dispersed cells were washed in complete medium to inhibit the trypsin then centrifuged to pellet the cells. The pellet was washed with PBS and suspended in complete medium at a concentration of 2.5×10^6^ cells/ml. 10-µl aliquots of cell suspension were deposited on the underside of the lid of a 10-cm tissue culture dish. The bottom of the dish contained 5-ml of PBS and served to prevent evaporation of the drops by forming a hydration chamber. Hanging drops were created by inverting the lid over the hydration chamber. Soluble Fn was added, where required, at a final concentration of 30 or 300 µg/ml. The drops were incubated at 37°C, 5% CO_2_, and 95% humidity for 3 days allowing cells to coalesce, form sheets, and rearrange to form spheres. At day 3, the spherical aggregates were transferred to 10-ml shaker flasks (Belko Glass, NJ) in 3-ml complete media and placed in an orbital shaker at 110 rpm for another 2–3 days. Accordingly, during the first 3 days in hanging drop cultures, aggregates possess liquid-like properties since their constituent cells possess the ability to move relative to one another and rearrange into spheres. Only after 3 days in culture did some subset of aggregates begin to exhibit elastic-solid-like properties.

#### b) Tissue surface tensiometry

Detailed methods describing TST are described in [8], [9]. Briefly, spherical aggregates ranging in size from 450–650 µ in diameter were transferred to the inner chamber of the tissue surface tensiometer and positioned on the lower compression plate (LCP). The inner chamber contained pre-warmed, de-gassed CO_2_-independent medium (Gibco-BRL, NY) supplemented with 10% FCS and antibiotics. The upper compression plate (UCP), attached to a nickel-chromium wire, was then positioned above the aggregate and connected to a Cahn electrobalance. The weight of the UCP was zeroed to establish a pre-compression UCP weight baseline. In order to minimize adhesion of cell aggregates to the compression plates, both the lower and upper plates were pre-coated with poly-2-hydroxyethylmethacrylate (poly-HEMA, Sigma, MO), a polymeric material to which cells do not adhere [41]. Compression was initiated by raising the LCP until the aggregate became compressed against the UCP. Adjusting the height of the LCP controlled different degrees of compression. The force with which the aggregate resisted compression was monitored by the Cahn recording electrobalance. Aggregate geometry was monitored through a 25x Nikon dissecting microscope equipped with a CCD video camera and connected to a Macintosh Power PC computer. Images of aggregates were captured, digitized and their geometries were analyzed using NIH Image software (Bethesda, MD). Each aggregate was subjected to two different degrees of compression, the second greater than the first. Measurements of aggregate geometry and the force of resistance to the compressive force were then utilized in the Young-Laplace equation [42], producing numerical values of apparent tissue surface tension (σ).

#### c) Confirmation of aggregate liquidity

Liquids have a true measurable surface tension whereas elastic solids exhibit what is sometimes referred to as an “apparent” surface tension. More precisely elastic solids exhibit a surface free energy per unit area, which although has the same units as liquid surface tension, is more complicated. A major distinction between liquids and elastic solids is that for liquids, the surface free energy is independent of area, whereas elastic solids exhibit an area-dependent surface free energy. Here we use the term sigma (σ) to denote both liquid and elastic surface free energy/unit area. In order to assess aggregate mechanical properties, we calculate aggregate response to an applied force and determine whether aggregates possess area-independent (liquid) or area-dependent (solid) surface free energy. Accordingly, the calculated free energy per area of a liquid aggregate, when subjected to two successive compressions, the second (σ_2_) greater than the first (σ_1_), will remain constant. In such aggregates the ratio of σ_2_/σ_1_ will be equal to 1 and will be less than the ratio of the initial force applied at each successively greater compression (F_2_/F_1_). In contrast, because the free energy of an elastic solid is area-dependent, it will increase proportionately to the imposed stress [7], [8], [9]. An elastic aggregate will obey Hooke's law and surface energy/area will increase proportionately to the applied force. For elastic aggregates the ratio of σ_2_/σ_1_ will not be equal to 1 but will instead approach the ratio of F_2_/F_1_. For liquid aggregates, the surface energy/area will also be independent of aggregate size. Only measurements in which surface energy/area is independent of the applied force and size were used to calculate average σ (true surface tension) for each of the α5β1 integrin-expressing cell lines reported in Fig. 3C–E and Fig. 4.

#### d) Statistical analysis

To compare the relationship between fiber size and sFn concentration or α5β1 receptor expression, a two-way ANOVA and Bonferroni's post hoc test were used to analyze the data (Table 1). A similar analysis was used for testing fiber density (Table 2). To test the relationship between σ_1_ and σ_2_ for aggregates within each clone, a paired t-test was used to determine whether σ_1_ and σ_2_ differed significantly (Table 3). A two-proportion Z test was performed to determine whether the ratios of σ_2_/σ_1_ differed significantly from F_2_/F_1_ (Table 3). Linear regression analysis was used for comparison of σ and volume (Fig. 3C–E). A one-way ANOVA and Tukey's Multiple Comparison Test were used to compare mean σ between the four cell lines (Fig. 4). Two-way ANOVA and Bonferroni's MCT were used to analyze compaction data in Fig. 5. A Student t-test was used to compare sFn depletion between HH and M aggregates (Fig. 6A). An ANOVA and Tukey's MCT were used to compare sFn depletion over time (Fig. 6B).

**Table 3:** We validated the true-liquid nature of the aggregates by comparing the ratio of surface tension (σ_2_/σ_1_) to the ratio of initial applied force (F_2_/F_1_) and the invariability of σ_1_ and σ_2_ for two successive compressions.

	σ_1_ (dynes/cm) ± s.e.m.	σ_2_ (dynes/cm) ± s.e.m.	σ_1_ vs σ_2_ P	σ_2_/σ_1_	F_2_/F_1_	σ_2_/σ_1_ vs F_2_/F_1_ z (α = 0.05)
L	5.5±0.4	5.8±0.4	0.59	1.05	1.52	−6.1
M	10.4±1.0	10.6±1.0	0.89	1.02	1.54	−4.6
H	5.3±0.4	5.8±0.4	0.39	1.09	1.56	−2.6
HH	5.4±0.4	5.7±0.2	0.51	1.06	1.51	−3.3

We show that these aggregates behave as true liquids since the ratio of force to the ratio of sigma is significantly different (two-Proportion Z test), and that σs calculated for different degrees of compression are not (Student paired t-test). The critical value of z at α = 0.05 is −1.65. As seen in Table 3, z values were smaller than the critical z value, indicating that the ratios of σ and F are significantly different.

### Compaction assay

Hanging drop cultures were prepared as described above but at concentrations of either 2.5×10^6^ cells/ml or 5.0×10^6^ cells/ml generating aggregates containing 25,000 tor 50,000 cells. For each cell line expressing different levels of α5β1 integrin, sets of 10 hanging drops were made and incubated for 2, 3, 4, and 5 days. At each time point, high contrast images of aggregates were capture using a bright-field microscope (Nikon Eclipse TE300) and IPLab imaging software (Scanalytics Inc., Fairfax, VA). Size measurements were made using ImageJ Software (N.I.H., Bethesda) by hand-drawing a perimeter around each aggregate and calculating area. Data sets were processed and graphed using Microsoft Excel to calculate mean, standard deviation, and SEM.

### Measurement of sFn depletion by ELISA

In order to measure the rate of soluble fibronectin depletion by aggregates in hanging drops, medium was collected after 3, 4, or 5 days in culture. Approximately 25,000 cells were suspended in Fn-depleted medium to which 30 µg/ml of rat plasma Fn was added. The assay was temporally designed so as to collect samples on the same day. Medium from 20 drops was pooled and centrifuged at 14,000×g for 10 minutes at 4°C. For ELISA, wells of Immuno 96 MicroWell™ Plates (Nunc™, Nalge Nunc International, Rochester, NY) were blocked for 1 hour with 200-µl of 1x PBS/0.1% BSA then washed four times with PBS/0.05% Tween 20. A rat soluble fibronectin standard curve was prepared from a 500 µg/ml stock (Calbiochem, Los Angeles, CA) diluted in Dulbeco's Modified Eagle Medium (DMEM). 100-µl triplicates of the standard curve, duplicates of each sample, and DMEM (as a blank) were loaded into the blocked wells and incubated at room temperature for 45 minutes. The plate was then washed four times with PBS/0.05% Tween 20, whereupon 100 µl of a 1∶4,000 dilution of a biotinylated primary rabbit polyclonal fibronectin antibody (Abcam 6584, MA) was added to each well and incubated for another 45 minutes at room temperature. After washing 4 times with PBS/0.05% Tween 20 wells were incubated with 100 µl of a 1∶10,000 dilution of an HRP-conjugated streptavidin (Pierce, Illinois). After washing, 100 µl of 3,3′,5,5′ tetramethylbenzidine (TMB) substrate (Sigma St. Louis, MO) was added to each well and incubated with shaking for 5 minutes. Color development was achieved by adding 50 µl of 2 M H_2_SO_4_. Optical density of the standards and samples was measured at 450 nm using an iMark Microplate Absorbance Reader (Biorad, Hercules, CA) and concentrations were quantified using standard methods.

### Cell sorting assay

Near-confluent monolayers of α5β1-transfected clones were dissociated in trypsin-EDTA (TE). Dispersed cells were washed in complete medium to inhibit the trypsin then centrifuged for 1 min at 50× g to pellet clumps. Single cell suspensions were stained with either PKH26 Red Fluorescent General Cell Linker or with PKH2 Green Fluorescent General Cell Linker (Sigma, St. Louis MO) as recommended by the manufacturer. Stained cells were mixed in equal proportion and placed in hanging drop culture in the presence of standard tissue culture medium, or in medium containing 30 or 300 µg/ml rat plasmam fibronectin, as previously described. Hanging drops were incubated for 2–3 days to allow aggregates to achieve stable equilibrium configurations. Aggregates were fixed in 2% paraformaldehyde in PBS and imaged by epifluorescence microsocopy.
